# Diagnosing intuition: a phenomenological account of intuitive knowledge in clinical practice

**DOI:** 10.3389/fpsyg.2025.1623145

**Published:** 2025-09-16

**Authors:** Giulia Lanzirotti

**Affiliations:** Department of Philosophy, Sociology, Education and Applied Psychology (FISPPA), University of Padova, Padova, Italy

**Keywords:** intuition, medicine, phenomenology, know-how, *categorial intuition*, hermeneutical intuition

## Abstract

This paper inquires into the nature of clinical intuition through the lens of phenomenology. Although intuition plays a significant role in diagnosis, its nature remains controversial, frequently portrayed as vague, irrational, or unreliable. Drawing on the phenomenological philosophies of Edmund Husserl and Martin Heidegger, I shall argue that intuition is not a mere emotive response but a structured and interpretive form of knowledge. After reviewing clinical literature and introducing Dreyfus’s model of skill acquisition, the paper examines Husserl’s *categorial intuition* and Heidegger’s *hermeneutical intuition*. These two notions challenge the dichotomy between intuition and rationality by revealing the multifactorial nature of experience. Finally, the paper applies these insights to the phenomenon of praecox feeling in schizophrenia diagnosis, demonstrating how phenomenology can illuminate the complex structure of intuitive experience in diagnostic procedures.

## 1 Introduction

Across all its branches, medicine is grounded in a canonized yet continuously evolving body of theoretical and practical knowledge that provides a common foundation for any clinical practice. Alongside this official shared *corpus*, clinicians—including psychiatrists, nurses, and other medical staff in hospitals and family doctors—recognize the presence of cognitive processes and epistemic approaches in diagnoses that go beyond standardized methods.

In recent years, a growing body of literature has explored the role of intuition in clinical contexts. These studies seek to better understand how clinicians employ, rely on, and assess intuitive judgments in both common and uncommon situations. While some researchers point out that intuition remains under-theorized despite its pervasive presence in clinical practice, others highlight that it remains controversial. Intuition is often described as difficult to define, understand, or evaluate, particularly regarding its epistemic legitimacy, practical function, and reliability. Given its slippery nature, many studies seek to clarify the value of intuition by examining its potential benefits and risks, both in general and across specific medical specialties (Van den Brink et al., 2019). Or else, they ask whether intuition is a tool that enhances and refines diagnostic accuracy, clinical decision-making and patient understanding, or, on the contrary, it is an unreliable resource, shaped by personal biases and prone to mistakes stemming from unquantifiable or flawed comprehension (Woolley and Kostopoulou, 2013). Finally, ascertaining the features of intuition is central to comprehending whether physicians can learn it.

To tackle these questions, researchers have pursued diverse strategies to define and investigate intuition. Most commonly, intuition is positioned in contrast to rational or analytical reasoning, described via synonymous terms such as “gut feeling” or “praecox feeling.” Generally, empirical studies have aimed to elucidate its meaning through clinician interviews, narrative accounts, and even investigation of neurological correlates of intuition (Srivastava and Grube, 2009; Lieberman, 2000; Naqvi et al., 2006).

Nevertheless, these approaches rarely engage with philosophical analysis of intuition, which might shed light on its underlying structure and clarify what truly happens when we experience what is commonly and often ambiguously referred to as “intuition.”

This paper argues that intuition is far from being a mere irrational feeling or emotive hunch. Drawing on the phenomenological insights of Edmund Husserl and Martin Heidegger, I aim to show that intuitive experience has a complex and structured nature. I do not intend to propose an exhaustive analysis of clinical diagnosis or a complete philosophical account of intuition. Indeed, the term itself encompasses a wide range of characterizations, including its everyday meaning—often assumed in medicine as a sudden insight or “gut feeling”—to diverse philosophical conceptions from Plato onwards. While being cautious in suggesting a complete overlap between the nature of intuition in clinical practice and intuition in phenomenology, I shall argue that phenomenological analysis can shed light on what is at stake when clinicians refer to “intuition.” Every day use tends to highlight only the outcome—a sudden *eureka* or an emotionally driven immediate response—without revealing the underlying process, thereby reducing intuition to an irrational mode of understanding. Rather than being an exceptional flash of insight, intuition in phenomenology represents the ordinary, continuous way in which we grasp and make sense of our experiences. Moreover, phenomenology represents the philosophical attempt to overcome those dualisms brought by Western philosophy. Under an ontological or epistemological view, phenomenology has tried to identify the relational structure of experience and to challenge the rigid opposition, such as that one intuition/rationality, to demonstrate the articulation of our experience in space and time. Contrary to the dominant view of intuition as vague feeling or an unstructured hunch, phenomenology reveals it as an experience shaped by multiple integrated dimensions—perceptual, conceptual, affective, and cultural—making it difficult to reduce it to an irrational grasp of reality.

I shall begin with a brief overview of clinical literature on intuition, including its relation to learning and expertise. Hubert Dreyfus’s model of skill acquisition will serve as a transitional framework, portraying intuition as a form of embodied “know-how” that emerges from situated practice. Then, I will turn to the core features of Husserl’s doctrine of *categorial intuition* and Heidegger’s notion of hermeneutic intuition. Husserl’s account highlights that we can have an intuitive grasp not only of perceptual contents but also of categorial structures, such as relations, unities, or logical forms. Heidegger, drawing on Husserl, develops this insight further by problematizing the distinction between perceptual and *categorial intuition*, and by situating intuition within the broader horizon of language, habits, and culture.^1^

Both accounts conceive intuition as a direct, yet one that is inseparable from meaning, structure, and interpretation. These phenomenological models challenge the simplistic dichotomy between rational analysis and intuitive response.

Finally, I consider the clinical phenomenon of the *praecox feeling*, an early diagnostic intuition reported in the recognition of schizophrenia. This case will function as a focused application of the concepts developed throughout the paper, illustrating how phenomenological insights into intuition can inform a richer understanding of diagnostic experience. My overarching claim is that clinical intuition is best understood not as a mysterious faculty, but as a structured, interpretive form of knowing.

## 2 How to describe intuition in the medical world

Although a unified, technical, and formal definition of intuition in clinical practices does not exist, there is a broadly shared preliminary understanding of it. In research studies, intuition is (a) contrasted with rational reasoning, (b) associated with various related terms, and illustrated through clinicians’ narratives and examples.

### 2.1 Definition by contrast

It is widely recognized that clinical reasoning operates through a dual-process framework, which encompasses both intuitive and rational forms of cognition. Within this model, literature distinguishes between non-analytical (intuitive) reasoning and analytical reasoning as two opposing processes. Intuition is characterized as non-analytical, fast, automatic, effortless, and often emotion-based, whereas analytical reasoning is described as deliberate, rational, slow, sequential, effortful, and demanding (Van den Brink et al., 2019). Other studies in the nursing context stress that clinical intuition is a holistic knowledge derived through synthesis rather than through analysis (Chilcote, 2017).

Other definitions frame intuition as a form of subconscious and implicit knowledge vs. a conscious, effortful, and thoughtful reflection. In this perspective, intuition is described as “thoughts that come to mind without apparent effort” or the process of “making judgments without any awareness of reasoning” (Van den Brink et al., 2019, p. 2). Even more drastically, some scholars argue that intuition is a process that is “fast, a-logical, and inaccessible to consciousness” (Lieberman, 2000).

However, this rigid division between two types of reasoning—intuitive and analytical—risks oversimplifying the reality of clinical decision-making. In actual practice, reasoning processes are far more complex and intertwined. In fact, as some studies hold, “decision-making processes are partially driven by emotion, imagination, and memories crystallized into occasional insights” (Sinclair and Ashkanasy, 2005, p. 354). This observation suggests that intuition and rationality are not mutually exclusive but rather deeply interwoven in ways that defy a purely dichotomous model.

### 2.2 Definition by synonyms or semi-synonyms

Another common strategy for defining intuition is to use synonyms or semi-synonyms. Intuition can be thought of as “first impression” or “insights,” although these terms must be distinguished. For some scholars, insight represents a sudden awareness of the logical relations between problems and their answers, while intuition represents simply an impetus or a tacit hunch (Lieberman, 2000, pp. 100–111) (Zander et al., 2016).

Much more often and consistently, intuition is equated with *gut feelings*—that is, “the visceral feelings that can inform diagnostic decisions,” an unconscious feeling “not reducible to cause-and-effect responses” (Van den Brink et al., 2019, p. 260). *Gut feelings* indicate that “pre-verbal process that cannot be explained: ‘You do not have a cognitive explanation for it but you do feel it’” (Vanstone et al., 2019, p. 23). It seems that the expression is not a metaphorical device, as clinicians report that their intuitive moments are often accompanied by actually felt bodily sensations (Chilcote, 2017, p. 197). Hence, *the gut feelings metaphor links intuition to physical sensations, thereby emphasizing* its non-logical or pre-logical nature. By framing intuition in such corporeal terms, this metaphor implicitly revives the traditional mind–body dichotomy: intuition is associated with the body, emotion, and instinct, while rationality is situated in the mind, intellect, and conscious control. In this way, the metaphor is not merely illustrative but carries significant conceptual implications, enhancing the idea of intuition as a purely irrational element in clinical diagnoses.

More specifically, *gut feelings* can manifest as an alteration of normality, as “a sense of alarm and a sense of reassurance.” They act as a signal that something may be wrong—or, conversely, that everything appears to be in order, and may suggest “to slow down and switch to analytical reasoning” (Sinclair and Ashkanasy, 2005, p. 197; Witteman et al., 2012).

Among possible related terms, there is a specific kind of intuition with a particular history. In psychiatry, clinicians have talked about *praecox feeling*, a subjective experience of strangeness and bizarreness (Greenhalgh, 2002) that the psychiatrist perceives during a clinical meeting with a patient affected by schizophrenia. We will discuss it in section 5 in more detail, but let us anticipate that *praecox feeling* has been evaluated as a helpful tool for diagnosing schizophrenia, a pathology that does not show specific symptoms. Preacox feeling, hence, represents a resource that should be learnt. This last observation leads us to the following subsection.

### 2.3 Intuition as skills

Other definitions of intuition emphasize its connection to expertise, equating it with “the art of medicine” or “clinical acumen” (Vanstone et al., 2019, p. 260). For some clinicians, intuition represents “the mark of an expert” [or, vice versa, “as mere guesswork, unnecessary in the age of evidence-based medicine” (Stolper et al., 2011, p. 60; Stolper et al., 2009)], or a “kind of ability to see through clients, something that clinicians who favor evidence-based practice should have nothing to do with” (Witteman et al., 2012, p. 19).

The value and trust placed in intuition seem to vary according to a clinician’s level of skilled experience. In this regard, participants in some studies have mentioned “experience as the most important determining factor, more specifically ‘on-the-job experience’ and learning from one’s own mistakes. The less experienced a physician is, the more analytical his/her approach will be.” They indicated that “younger doctors do not, and according to some should not, trust their gut feelings as much and will therefore consult a specialist-tutor for further guidance” (Van den Brink et al., 2019). Mental health care studies show that highly experienced clinicians tend “to rely less on deliberate analysis and more on pattern recognition and encapsulated knowledge and routine” (Witteman et al., 2012, p. 21; Rikers et al., 2000; Witteman and van den Bercken, 2007). This raises a key question: *can intuition or intuitive acumen be learned?* While opinions differ, most agree on the possibility of learning: “it can be developed, not so much at college as in clinical practice and through supervision, especially in client-centered training. More specifically, ‘You can learn to take it seriously’, to use it properly and to trust it, for example, by looking back at videotapes of your own sessions with a supervisor” (Witteman et al., 2012, p. 24). These lines can help us appreciate how pivotal interpersonal relationships are. Intuition is not developed through rule-based, theoretical knowledge alone but emerges from situated, relational, and concrete experiences. To enrich intuitive skills, it is necessary to engage with patients in their concreteness and individuality and establish a confrontation with an expert supervisor. Practice, then, is key.

These last considerations highlight how intuitive responses tend to become more robust as clinical experience becomes more skilled and holistic. In this sense, intuition is not only a response reserved for exceptional situations, but it continuously operates, involving “non-conscious scanning of internal (in memory) and external (in environment) resources” (Sinclair and Ashkanasy, 2005). So, w*hen does intuition play a role?* Clinicians identified three moments: “1. directly at the first contact; 2. when in doubt or uncertain; and (3) always.” (Witteman et al., 2012, p. 23).

To conclude, it is worth noting a couple of details. Intuition is more positively evaluated in specialities (1) like emergency medicine, where prompt, life-saving decisions are often required; (2) like pediatrics, psychiatry, nursing contexts, in which subjective, interpersonal relationships have a major role compared to more strictly pure medical practices (assuming such a purity may exist) (Van den Brink et al., 2019).

## 3 Dreyfus’ intuition as *know-how*

Before turning to Husserl and Heidegger’s phenomenological account of intuition, I would like to briefly consider Hubert Dreyfus’s influential interpretation of expertise. As we mentioned, one of the central questions surrounding intuition is how it can be acquired. Dreyfus’ model of skill acquisition has been widely used to explain how intuitive responses emerge through a learning process, also in contexts like clinical practice (Benner, 2004).

In his career, Dreyfus has engaged with the phenomenological and hermeneutic tradition and the critique of artificial intelligence. Thanks to his understanding of phenomenology, especially Heidegger and Merleau-Ponty, he developed a specific expertise and skills acquisition model. In *Mind Over Machine* (Dreyfus, 1986), he outlines five sequential stages of skill development: *Novice*, *Advanced Beginner*, *Competence*, *Proficiency*, and *Expertise*. To account for this progression, Dreyfus draws on the classical distinction between *knowing-that* and *knowing-how* (see Ryle, 1945; 2009). While *knowing-that* refers to propositional, explicit knowledge (I know that 2 + 2 = 4), *knowing-how* describes embodied, practical skills (I know how to swim, I know how to play tennis). As Dreyfus holds, “all of us know how to do innumerable things (…) like bike riding” that “cannot be reduced to ‘knowing that’” (Dreyfus, 1986, p. 16).

According to Dreyfus, we are not fully aware of the extent to which we rely on this tacit, embodied.

*know-how* because we take it for granted and we do not appreciate “the extent to which it pervades your activities.” *Know-how* skills, of course, are not innate but learned. For Dreyfus, the learning process involves a shift from rule-guided, abstract reasoning (*knowing-that*) to context-sensitive, qualitative experiential understanding (*knowing-how*) in which the learner goes through “qualitatively different perception of his/her tasks and mode of decision-making” (Dreyfus, 1986, p. 19).

While there is no need to detail all five stages, I will focus on the novice and the expert. The novice learns features and explicit rules as context-free and follows them, not being taught that rules may be violated in certain situations. For example, a novice chess player learns how to assign points to pieces and follows the rule of exchanging a piece for the opponent if the total of pieces captured exceeds that of pieces lost, while, maybe, on certain occasions, this is not a good deal (Dreyfus, 1986, p. 22). On the contrary, the expert does not need to make conscious deliberative judgments as skills have become part of him (Dreyfus, 1986, p. 30). Dreyfus maintains that when things are proceeding normally, “experts do not solve problems and do not make decisions: *they do what normally works*” (Dreyfus, 1986, p. 31). Hence, experts move from rule-based, analytical thinking to a more holistic, intuitive grasp of situations. This form of intuition is not detached from learning but rather is the product of it, the result of infinite situated encounters where perception, bodily movement, and practical judgment are combined. For Dreyfus, intuition is synonymous with *know-how*; it is not wild guessing or supernatural inspiration, but a sort of ability we all use in our everyday tasks, and that for Dreyfus has been neglected by the traditional understanding of rationality (Dreyfus, 1986, pp. 28 and 29).

Thus, intuition, understood as *know-how*, can be learned. We can observe that Dreyfus’s conception of intuition overlaps with many descriptions found in the medical literature, particularly its portrayal as a fast, immediate, and unreflective skill. Nonetheless, he places a stronger emphasis on the idea that intuition is not merely rare or exceptional, but a constantly operating aspect of skilled performance (Vanstone et al., 2019). This characterization distinguishes intuition as *know-how* from the idea that gut feelings indicate a visceral experience. Unlike bodily sensations that communicate an alerting sign, *know-how* often goes unnoticed because it is rooted in our daily routine and habits, and we take it for granted. Conceiving intuition as *know-how* uncovers a dimension of intuition that may challenge a rigid separation between analytical and non-analytical reasoning.

Dreyfus’ account encourages us to reconsider the dual-process model not as a binary opposition but as a dynamic interplay, where intuitive expertise plays a central and constant role in decision-making. However, Dreyfus himself does not really make this step and remains anchored to a dual understanding of experience that resonates with the dual-process model discussed in section 1.

In several of his papers (Dreyfus, 2006; Dreyfus, 2014; Dreyfus, 1980; Dreyfus, 2005; Dreyfus, 2007a,b; Dreyfus, 2002), Dreyfus has distinguished between two fundamental dimensions of experience. The more original and primary of these is the domain of practice—skills, bodily movements, and what he terms *embodied coping*—or, in other words, the realm of *knowing-how*. In this mode of engagement with the world, we move, act, and use tools within a context of habits and practical familiarity without deliberate judgment or reflection.

Experts, be they car drivers, chess masters, or tennis players, often perform their actions without conscious thought or intellectual *detour*. As already hinted, a chess master may not even conceptualize the fact that his hand is choosing a piece to move or calculate his strategies by pondering all possible alternatives. According to Dreyfus, such actions occur without the involvement of mental representation, explicit conceptual processing, or linguistic mediation. Alongside this practical mode of being, he identifies another level of experience in which we speak, judge, think, and operate with conceptual content.

To articulate the distinction between these two modes, Dreyfus draws directly from Heidegger’s terminology in *Being and Time*, particularly the opposition between *Zuhandenheit* (readiness-to-hand) and *Vorhandenheit* (presence-at-hand) (Dreyfus, 1990; Dreyfus, 2006; Dreyfus, 2014; Heidegger, 1962). As Heidegger puts it, we use the hammer to hang a picture without evaluating our equipment. However, as soon as the hammer breaks up, we may stop, observe it, and judge that it is too heavy. The shift between these two attitudes is the change between what Dreyfus describes as our absorbed, skilful engagement with the world and our detached, objectifying stance toward it.

Once again, the distinction between these two attitudes closely mirrors the contrast between non-analytical and analytical reasoning frequently discussed in medical literature. On one side, non-analytical reasoning is grounded in individualized, experiential practice, bodily sensations (such as gut feelings), and context-dependent perception. On the other hand, analytical reasoning involves deliberate reflection, explicit judgment, and slow, systematic thinking. Nonetheless, as previously mentioned, Dreyfus’s account of expertise and intuition constitutes a structured learning process that involves conceptual acquisitions and progressive integration of experience. Far from being a mere irrational feeling, expertise, across all disciplines, also emerges through a slow assimilation of rules and theoretical knowledge. More generally, the opposition between perception and concepts—as if they belonged to entirely separate registers—requires critical reassessment. Phenomenology offers valuable insight here. Husserl and Heidegger challenge the dichotomy between raw perception and conceptual thought through the notions of categorial and *hermeneutical intuition*. These concepts reveal how perception is always shaped by meaning and how intuition can be both immediate and conceptually structured. Despite his phenomenological background, Dreyfus overlooked these intuitions, thereby reinforcing the very dualism that phenomenology seeks to dismantle.

## 4 Phenomenological intuition

Discussing intuition in philosophy is a lifelong enterprise, as it represents one of the discipline’s most fundamental concepts. For the purposes of this paper, however, I will considerably narrow the focus to the notion of *categorial intuition* and *hermeneutical intuition*. These concepts will allow us to see that immediate experience is not limited to pre-logical or purely perceptual elements, but is also structured by the conceptual dimensions.

### 4.1 Categorial intuition

Intuition occupies a central role in Husserl’s phenomenology. In its primary sense, intuition denotes an immediate form of knowledge in which the object is given directly to consciousness without being mediated by subjective inference or conceptual construction (Witteman et al., 2012, pp. 175–177). As Husserl famously asserts in *Ideas*: “Immediate seeing, not merely sensuous, experiential seeing, but seeing in the universal sense as an originally presentive consciousness of any kind whatever, is the ultimate legitimizing source of all rational assertions” (Husserl, 1982, §19).

In the *Logical Investigations*, Husserl expands this notion beyond mere sense perception. In the *Sixth Investigation* (Husserl, 1970), he introduces the doctrine of *categorial intuition*, a special kind of intuition meant to explain the relation between the expressive act and its intuitive fulfillment (Husserl, 1970). In other words, the original doctrine of *categorial intuition* aims to justify how our judgments, which express our beliefs as “that crow is black” or “that chair is yellow,” can be true and have a correspondence with the actual fact that the crow is indeed black and flying away.

For our purposes, the doctrine of *categorial intuition* represents the attempt to bridge two seemingly separate domains: that of perception and that of intellect—which we have already seen in Dreyfus and the distinction between intuitions/gut feelings and reflective thinking.

Take the example of the statement “that crow is black.” When we look at the world, we may indeed see a crow and the color black. However, we do not see the “that” or the “is”—namely, the demonstrative and the copula. Similarly, we would not see any other conjunctions, prepositions, and other syntactic components as part of what is given in perception. In any judgment, we find nominal parts (nouns like “crow” and predicates like “black”) and “formal,” “syntactic,” or “categorical” components of language (“this,” “is,” and”). Syntactic parts are crucial as they have the function to connect all words and form a meaningful sentence that can be evaluated as true or false.

However, if the nominal parts can be directly fulfilled by corresponding perceptions (“bird” from the actual perceived bird), Husserl observes that syntactic forms “can find nothing that ever could fit them in perception or acts of like order” (Husserl, 1970, p. 276). Hence, while we visually apprehend the crow, when we judge that the crow is black, we are actively adding something more. It seems that linguistic connections belong to us, our minds, and our language, and cannot be experienced in the worldly domain. This leads Husserl to ask, what does fulfill these categorial forms? (Husserl, 1970, p. 271).

There is, then, a *surplus* in judgment—something more than what is directly given in sense perception—that demands explanation. One possible response is to argue that these logical forms belong not to the world, but to the mind, i.e., as mental operations such as combining, comparing, and abstracting ideas (Locke) or *a priori* forms of intellect (Kant). On this view, when we judge that the bird is black, our intellect synthesizes the perceptual content with the necessary categorial forms to shape a complete, meaningful judgment.

But Husserl pushes further. Even words (nominal parts) that appear straightforwardly referential–like “black”—do not perfectly coincide with the color as it appears in experience. There remains a *surplus* of meaning not confirmed by the appearance alone. A black crow is a crow that *is* black; white paper is paper that *is* white. The form “is” recurs, even when hidden. And Husserl asks: is not even the noun “crow” already shaped by a categorial structure? (Husserl, 1970, p. 273). Thus, the entire sentence exceeds what is given in mere perceptual experience.

Husserl’s move is to reject and overturn the Lockean and Kantian solution. For Husserl, categorial forms and intellectual categories (as conceived by Kant in the *Critique of Pure Reason*) are not psychological forms. When we say that the “crow is black,” we are not projecting our *a priori* intellectual forms, but we are thing-directed. For Husserl, we see more than sensible data: we can *see* the categorial articulation since categories are already embedded within experience itself (Costa, 2003).

This challenges traditional dichotomies. If we interpret intuition as a kind of embodied know-how, as Dreyfus suggests, or through the rigid opposition between analytical and non-analytical reasoning, we risk overlooking a more intricate conceptual framework. In the medical context, when clinicians acknowledge the presence of a dual system of reasoning in their practices and decision-making (analytical and non-analytical), they often observe that these two modes interact and blend. What we are proposing here by means of *categorial intuition* is that intuition itself should be understood as a *blend—*a dynamic integration of elements that philosophical and clinical traditions have typically treated as distinct.

Unfortunately, comprehending how the *categorial intuition* works is not an easy task, even for Husserlian scholars. Putting it as simply as possible, Husserl distinguishes the simple or perceptual intuition from the *categorial intuition*. Simple intuition gives us direct, immediate access to its object, what Husserl describes as grasping something “in one blow” (Husserl, 1970, p. 283). We perceive the object in its concrete singularity, for example, “this individual crow”, without any other further articulations. While simple intuition is the foundation of knowledge, *categorial intuition* is founded upon it.

At first, we grasp the object in one blink as a whole without any further structure. All its parts are implicitly there but not yet perceived (Lohmar, 2002, p. 131). On this basis, thanks to the *categorial intuition*, we explicitly intend its parts and relations, such as those of conjunction, disjunction, generalization (Husserl, 1970, p. 282), that the singular object possesses: we do not see only this crow, but that the crow with its black wings being on the tree. Hence, we see the crow in its relations with its own parts, with other objects, and its context, which we can later make explicit by saying that the black wings or a yellow beak are parts of that crow on the tree. Hence, according to Husserl, we can see what in the sentence was represented by the syntactical part of language, which, in *categorial intuition,* finds its fulfillment instead of being forms of intellect.

To sum up, we can divide the functioning of the doctrine of *categorial intuition* into three steps (Lohmar, 2002). We see (a) the crow as a whole, (b) the crow in its constitutive parts, and (c) we judge that the crow is black and on the tree. We do not perceive all these features as unrelated or isolated but as a synthesis of a special perception (the *categorial intuition*), thanks to which we can express linguistically the judgment: “The crow is on the tree.” Notably, the analysis of these three moments reveals that they do not stand in mere juxtaposition; instead, they are articulated in a temporal process, as emphasized by Heidegger (Römer, 2013), and constitute a whole experience. The phenomenological inquiry shows that what we ordinarily conceive as intuition—a sudden stroke of genius—can, in retrospect, be analytically unpacked into distinct moments that form a coherent experience.

In Husserl’s work, *categorial intuition* has a specific role within Husserlian understanding of phenomenological science and knowledge. For this paper, it represents a notion to include and consider when deconstructing the idea of intuition as an irrational and emotion-related feeling. What we gain from Husserl is that our intuition registers all those structural elements that belong to the rational domain. However, Husserl’s distinction between simple perception and categorial perception still entails some sort of separation between an unstructured level of intuition, the categorial articulation of experience, and conceptual language.

This kind of separation seems to disappear in Heidegger’s interpretation of *categorial intuition*. Heidegger explains Husserl’s doctrine in one of his lectures on phenomenological thought (Heidegger, 1992) but engages with it in various moments of his philosophical career (Heidegger, 1962, 2002, 2010, 2012).

### 4.2 Hermeneutical intuition

In his lectures, Heidegger applauds Husserl as he recognized that intuition has a broader meaning: it is not merely sensory; it is not an exclusively sensory act, as it encompasses “any act in which we apprehend an object, whether sensory or non-sensory” (Wrathall, 2021, p. 431). In commenting on Husserl’s doctrine of *categorial intuition*, Heidegger points out that it shows that there is a simple apprehension of categories, agreeing with Husserl that these are given within experience and are not subject-related.

In line with Husserl, Heidegger postulates a direct grasp of a categorially structured worldly experience. However, Heidegger moves forward by claiming that simple perception is already intrinsically pervaded by *categorial intuition* (Dreyfus, 2007a, p. 60). Or, in other words, that our perceptions are already expressed, even more, are interpreted in a certain way” (Dreyfus, 2007a, p. 56). These additional observations mark the difference between Heidegger and Husserl’s understanding of *categorial intuition*. To unpack the import of Heidegger’s interpretation, we should question his specific use of all terms in play here: perception, expression, and interpretation. Without taking this road, we can stick to their ordinary meanings. In the above-mentioned claims, we appreciate how Heidegger shortens the distinction between a simple perception and a categorial one. The neat divide between a perception (“I see the crow”) and its expression (“I say I see a crow”) becomes increasingly blurred and may even disappear (Dahlstrom, 2001). This implies that when we see a crow, we do not attach the meaning “crow” to the perceived bird in a subsequent moment. For Heidegger, the possibility of experiencing a pure perception or having a pure, simple intuition is an abstraction (Dahlstrom, 2001, p. 85). According to this view, for instance, the idea of a gut feeling as a sheer bodily sensation is an abstraction and a mistranslation of a multifaceted experience.

What we actually see in one blow is a bird in its categorial relations and its meaning. According to Heidegger, we do not say what we see, but instead, we see what we say about a specific subject (Heidegger, 1992, p. 56). Furthermore, Heidegger talks about interpretation since what we perceive and say is always situated in a certain context, in space and in time, and in culture. Building on Husserl’s *categorial intuition*, Heidegger introduces his own concept of *hermeneutical intuition* (Heidegger, 2002), which suggests that our intuitions are not simply perceptions; they also involve a particular interpretative stance influenced by the specific cultural horizon we live in. Following Heidegger, we can use this example to illustrate the combination of perception, meaning, and interpretation (see Wrathall, 2021). When we enter a classroom, we see a desk. Immediately, without deliberation, we perceive it as such: our eyes perceive it, and we sit for the lecture. We do not first register a flat wooden surface and then infer that it must be a desk. In one blow, as Husserl would say, we see a desk that we know is a desk, not a counter or bedside table. Our perception is already filled with specific meanings. But now imagine someone unfamiliar with schools—someone who has never been a student or teacher. They might perceive the same object simply as a table. And someone from a completely different culture, where human beings do not use desks or tables, would see a strange thing, a den or shelter for the rain. The student would see a desk, and that is their interpretation; someone else could see it as a mysterious thing, and again, that would be their interpretation. This hermeneutical interpretative intuition is not a slow process, as a conceptual cognitive process would be traditionally characterized. In life, we are able to assign the meaning “desk” to what we perceive because we are immersed in a specific culture. We learn how to use things and words, and we move in a meaningful world that we perceive and think of in a given temporal and cultural habitual context. That is why we recognize a desk as a desk: not because we analyze it in a second moment, but because we already know how to live with it. Then, what we discover is the following: intuition, whether understood as gut feeling, know-how, or hermeneutic insight, represents a fast, direct, and immediate phenomenon. However, being fast and direct does not imply a lack of structure. As we have seen in phenomenological accounts of intuition, perceiving involves an entire web of interconnected elements. This includes structured perception, meaning, cultural context, and a learning process, to echo Dreyfus. We live in a world that becomes more and more familiar to us. We learn how to do many things, how to use language and communicate with other human beings. We share habits and rituals with other human beings from the same environment. In *hermeneutical intuition* or our know-how, our perception is infused with language, conceptualities, rules to be taught, and practical judgments. Even if not explicitly expressed, when we experience an intuition, all these elements inform our background and lead our thinking process and emotional reaction.

Our intuition thus emerges as both a product and a process, shaped tacitly by rational and ordering factors that shape our worldly experience in all its aspects. In *hermeneutical intuition*, our understanding (*Verstehen*) of experience is not a mere cognitive act added onto perception but a fundamental mode of our being-in-the-world. Understanding, in Heideggerian terms, is the existential condition that allows the world to appear as meaningful plethora of possibilities in which we are already engaged with.

To see how *categorial*/*hermeneutical intuition* may help us in reframing the intuitive experience in a medical context, we can make an example. As already mentioned, intuition can serve as an alert mechanism in medical practice and suggest a serious condition despite seemingly banal symptoms. A similar situation was reported in the following way: ““I got a call from a lady saying her three-year-old daughter had had diarrhea and was behaving strangely. I knew the family well, and was sufficiently concerned to break off my morning surgery and visit immediately.’ The clinician’s hunch led him to diagnose correctly, and treat successfully, a case of meningococcal meningitis “on the basis of two non-specific symptoms reported over the phone” (Greenhalgh, 2002, p. 395).

Here, we discuss the role of intuition in a rather extreme case. It seems odd or lucky that a doctor spotted a major disease from just a brief phone call reporting nonspecific symptoms. But actually, from what we can learn from phenomenology, we should isolate the phrase: “I knew the family well.” It is not secondary that the clinician was well familiar with the patients and their relatives. We might think the clinician was able to hear a change in the mother’s voice or knew that she would not call if not urgent. The clinician’s instantaneous and alert reaction does not explicitly dispose of all the features that served to arrive at a serious diagnosis. However, these features were implicitly operating. In a minimal arc of time (a phone call), the clinician’s reaction resulted from their acquired expertise, countless diagnoses, treatment cases, and practical routine. A novice could not be able to “jump” from diarrhea to meningitis. Together with this *know-how*, the refined intuition stemmed from interpersonal relationships with patients, which are vital in medical practices as they allow for establishing an individualized recognition of symptoms and an empathic personal knowledge of a singular patient. Generalized rules (symptoms of disease and their treatment) must be applied to a singular individual, and each individual has its peculiarity also in a general pattern of behavior. In this report, the clinician’s experience gathered all these aspects, and was expert enough to trust their so-called “gut.”

## 5 Praecox feeling as a case study

As previously mentioned, there is a particular form of intuition in psychiatry known as the *praecox feeling* that I would like to briefly analyse as a clearer case of *categorial intuition*.

First theorized by Rumke (1942), this sensation serves as a valuable diagnostic tool in the early identification of schizophrenia. *Praecox feeling* indicates a passive intuition (Moskalewicz et al., 2018), a sensation of strangeness and bizarreness, averted in front of the patient. This feeling is both vague and yet certain, difficult to translate into words, but surprisingly accurate in distinguishing schizophrenia from other types of psychosis. Some studies describe the *praecox feeling* as an atmosphere that arises in face-to-face interactions, often even before the patient talks, as it may emerge just from the observation of elements such as bodily posture and facial expression, and overall lack of emotional contact (Szuła et al., 2023).

In the history of psychiatry, the role of empathy and the importance of a close, interpersonal relationship with the patient in diagnosing schizophrenia have been highlighted by several psychopathologists, variously connected to phenomenology, such as Minkowski (2013), Binswanger (1955), and Jaspers (1997). All of them have stressed the possibility of directly recognizing schizophrenia. However, in recent times, the validity of the *praecox feeling* has been questioned, as the newest DMS guidelines privilege and recommend context-independent reasoning and a third-person perspective in psychiatric practices (Minkowski, 2013). Nonetheless, experts consider it a valuable instrument, especially given that the symptoms of schizophrenia are typically non-specific and no reliable biomarkers are currently available (Gozé, 2022).

Following a phenomenological approach, Gozè and Moskalewich examine the functioning of *praecox feeling*. They draw on the concept of *typification* from the late Husserl (1975), referencing Schwartz and Wiggins who describe *typification* as a tacit perceptual process in which we recognize a patterned form (*Gestalt*), even when information is incomplete. As Husserl notes, we never perceive an entire building, only a side of it, but this partial view refers us back to the building’s idea (*eidos*), allowing us to anticipate its unseen aspects. Similarly, Schwartz and Wiggins argue that *typification* enables clinicians to infer a patient’s personality from limited cues. According to Gozè, Husserlian *typification* rests on the assumption of a stable and predictable world. It links the unknown to the known, thus reducing unfamiliarity and surprise. But Gozè argues that *praecox feeling* is precisely nurtured by the experience of surprise and bizarreness that should be foregrounded (Ferencz-Flatz, 2014). In my understanding, *typification* resonates with the dynamics of Husserl’s *categorial intuition* and Heidegger’s *hermeneutical intuition*. Types emerge with habits and their connoted recurrence in our perceptual and practical life (Ferencz-Flatz, 2014). Like *hermeneutical intuition*, *typifications* are contextually constituted (Fernandez, 2016), and familiarity or unfamiliarity stems from a given culture. In the case of *praecox feeling*, I would argue that the psychiatrist needs to possess specific training in the discipline, habitual practice in patient care, and the ability to interpret bodily expressions or pathological speech. The sense of strangeness arises precisely because we are accustomed to particular patterns of behavior and language, shaped by our cultural background, shared education, and disciplinary formation. Hence, even bizarre feelings are grounded in habitual experience, as disruptions of it. Moreover, when a bizarre feeling becomes a starting point for diagnosis, it can itself be integrated into a habitual clinical schema. This is illustrated in Gozé’s own account of the *praecox feeling*, which he describes as temporal unfolding in three steps: (1) *Bizarre Contact*: The initial sense of strangeness is perceived vaguely, as an atmospheric or pre-reflective impression. This “oddness” may be felt by both laypeople and clinicians, even if they cannot yet identify its source. It belongs to the antepredicative, perceptual level. (2) *Typification Process*: The clinician begins to recognize that the strangeness does not stem from the surrounding context but originates from the patient themselves. This step involves pre-reflective *typification*, made possible through the psychiatrist’s professional experience and habitual sensitivity to certain clinical patterns. (3) *Reflexive predicative judgment*: Finally, the clinician attains a specific and conscious recognition of the experience. While the second stage operates at a pre-reflective but already structured (predicative) level, this third stage marks the moment when the impression becomes part of the diagnosis (Gozé, 2022). At the same time, the clinicians shall consider these feelings as potentially valuable for their assessments, while being careful not to rely solely on subjective impressions. In the case of *praecox feeling*—a kind of intuition specifically associated with schizophrenia—the temporal structure of the medical encounter offers space for reflection. This allows clinicians to examine their intuition critically and integrate it with objective, shareable scientific criteria. In this process, the *praecox feeling* becomes aligned with the final diagnostic conclusions, thereby functioning as a meaningful tool in clinical assessment.

The second step represents more distinctly how expert clinicians experience *praecox feeling*. However, drawing from Husserl and Heidegger, the first step is not purely sensory. Perceiving something as odd presupposes familiarity, and familiarity involves a complex interplay of perception and cognition shaped by one’s cultural and personal history. As laypersons, we would not recognize schizophrenia because we lack the relevant clinical knowledge. Nevertheless, our sense of something being “odd” would still arise from our own culturally shaped conception of what counts as strange. Crucially, as laypersons, we would not interpret this oddness diagnostically. Our encounter with a person with schizophrenia would not be framed by a diagnostic attitude. In contrast, for the clinician, the experience of bizarreness is already entangled with diagnostic interpretation. While it might be possible to analytically separate the sense of the bizarre from *typification*, in practice, they are inseparable. The clinician’s perception is informed by medical training and shaped by the goal of diagnosis. As a result, the bizarreness experienced by a clinician differs qualitatively from that of a layperson.

I shall emphasize that *gut feelings* and *praecox feelings* have often been discussed without sufficient attention to their rational and conceptual dimensions. Situating intuition or *praecox feelings* within a given context is not merely a matter of physical or cultural placement. Rather, “context” refers to a complex web of relationships—social, historical, cognitive, and institutional—that contribute to perception and meaning.

## 6 Conclusion

Intuition is a concept that bears a variety of definitions. In medical literature, it has mostly been characterized as an irrational feeling that clinicians may trust or not. To sum up, for literature intuition is: rapid, an unconscious process, context-dependent, comes with practice, cannot be reduced to cause-and-effect logic (Greenhalgh, 2002, p. 396), and a tacit knowledge (Polanyi, 2009).

Turning to phenomenology allows us to introduce a distinctive conception of intuition into the debate. Through *categorial* and *hermeneutical intuition*, we can think of intuition as exceeding standard definitions. Phenomenological accounts suggest that experience is neither purely perceptual nor entirely rational. Our perceptions are shaped and articulated by linguistic and cultural meanings, situated in context, and influenced by numerous forms of training. Fast, intuitive responses are not necessarily simple or purely instinctive. Familiarity, habit, and education make our everyday perceptions and cognitions more efficient. These habits and forms of training require the acquisition of highly specific and sophisticated rational understanding, linguistic competence, comprehension of rules, and more. All these capacities are operative within our intuitions and help us in framing how apparently ineffable sensations may lead to accurate diagnoses, like in the case of *praecox feeling*.

## Footnotes

^1^We shall consider that in Husserl’s works, the doctrine of *categorial intuition* appears explicitly only in the *Logical Investigations*. In his later writings, however—such as *Ideas I*, *Crisis*, and *Experience and Judgment*—Husserl further develops correlated notions such as *Wesensschau*, *Lebenswelt*, and *Typus*. These notions introduce the elements of the horizon, especially the cultural and habitual dimensions, which are not yet fully thematized in the account of *categorial intuition*. It is precisely these aspects that Heidegger’s idea of hermeneutic intuition seeks to capture. Nevertheless, both Husserl’s *categorial intuition* and Heidegger’s hermeneutic intuition are valuable here, since they specifically address the specific articulation between perception and cognition. This focus allows us to see how the ordinary understanding of intuition as an exceptional or mysterious act actually conceals the underlying structure of this articulation.
